# Integrated Amorphous Carbon Film Temperature Sensor with Silicon Accelerometer into MEMS Sensor

**DOI:** 10.3390/mi15091144

**Published:** 2024-09-12

**Authors:** Qi Zhang, Xiaoya Liang, Wenzhe Bi, Xing Pang, Yulong Zhao

**Affiliations:** 1 State Key Laboratory for Manufacturing Systems Engineering, Xi’an Jiaotong University, Xi’an 710049, China; zhq0919@xjtu.edu.cn (Q.Z.); liang_xiaoya@stu.xjtu.edu.cn (X.L.); biwenzhe@stu.xjtu.edu.cn (W.B.); 2 Electronic Materials Research Lab, Key Lab of Education Ministry, School of Electronic Science and Engineering, Xi’an Jiaotong University, Xi’an 710049, China

**Keywords:** amorphous carbon film, Temperature-Resistance Characteristics, MEMS, silicon accelerator, integrated sensor

## Abstract

Amorphous carbon (a-C) has promising potential for temperature sensing due to its outstanding properties. In this work, an a-C thin film temperature sensor integrated with the MEMS silicon accelerometer was proposed, and a-C film was deposited on the fixed frame of the accelerometer chip. The a-C film was deposited by DC magnetron sputtering and linear ion beam, respectively. The nanostructures of two types of films were observed by SEM and TEM. The cluster size of sp^2^ was analyzed by Raman, and the content of sp^2^ and sp^3^ of the carbon film was analyzed by XPS. It showed that the DC-sputtered amorphous carbon film, which had a higher sp^2^ content, had better temperature-sensitive properties. Then, an integrated sensor chip was designed, and the structure of the accelerometer was simulated and optimized to determine the final sizes. The temperature sensor module had a sensitivity of 1.62 mV/°C at the input voltage of 5 V with a linearity of 0.9958 in the temperature range of 20~150 °C. The sensitivity of the sensor is slightly higher than that of traditional metal film temperature sensors. The accelerometer module had a sensitivity of 1.4 mV/g/5 V, a nonlinearity of 0.38%, a repeatability of 1.56%, a total thermomechanical noise of 509 μg over the range of 1 to 20 Hz, and an average thermomechanical noise density of 116 µg/√Hz, which is smaller than the input acceleration amplitude for testing sensitivity. Under different temperatures, the performance of the accelerometer was tested. This research provided significant insights into the convenient procedure to develop a high-performance, economical temperature–accelerometer-integrated MEMS sensor.

## 1. Introduction

Due to the strict limits of the environment, space, and costs in the fields of aerospace, automotive, and environmental monitoring [1], the miniaturization, multifunctionality, and integration of sensors are crucial [2,3,4]. Many working scenes in narrow spaces, such as the machine’s hydraulic system, must detect multiple signals simultaneously. It will waste much space if many sensors with a single sensing function are used, so it is necessary to study small-sized and lightweight integrated sensors for multi-physical quantity testing [1,2]. Integrated sensors can combine different kinds of sensors based on the needs of the test environment, such as a pressure–acceleration sensor [1], a pressure–temperature-acceleration sensor [2], and a pressure–humidity sensor [3] et al. In 2007, our research group proposed an SOI silicon micro-multifunctional sensor that integrated accelerometer, pressure, and temperature, with a chip size of 4 mm × 6 mm × 0.9 mm, and achieved 3-axis MEMS accelerometers [4,5,6]. Amal Z. Hajjaj et al. proposed a new triple sensing scheme via nonlinear weakly coupled resonators for mass, stiffness, and acceleration sensing by simultaneously tracking the first three vibration mode resonance frequencies of weakly coupled resonators [7]. Zhaozong Meng et al. developed multisensor augmented UHF RFID tags integrating temperature, humidity, and illuminance sensors for wirelessly powered sensing and communication [8]. The above sensors are used for the simultaneous detection of different physical quantities and have a wide range of potential applications in these areas due to their small size and low unit cost.

Loose connections in machines’ pipelines seriously affect the working performance and service life. The looseness of the pipeline is caused by abnormal vibration of the hydraulic pipeline and high temperature at the fault. Since the hydraulic system has many pipelines and the test space is small. The traditional resistance temperature detector (RTD) is usually installed in the form of a probe, so the simultaneous installation of temperature sensors and vibration sensors will take up much space. However, when integrating these two kinds of sensors together, the weight of the package can also be reduced, making it both lightweight and space-saving. We integrated temperature sensors with accelerometers, using thin film deposition technology and bulk micromachining technology to fabricate a multisensor chip on a (100) SOI chip. The temperature-sensitive material is an amorphous carbon film, and the piezoresistive material is p-type silicon. Compared with other piezoresistive materials, p-type silicon has a higher piezoresistive coefficient [9,10], and the SOI chip substrate can withstand temperatures ranging from 0 to 300 °C [11,12,13].

Commonly used temperature-sensitive materials are metals (copper, silver, platinum, et al.) and metal oxides (aluminum oxide, titanium dioxide, tin dioxide, et al.). However, metal thermistor temperature sensors like precious metal platinum are expensive to manufacture, and metal oxide temperature sensors are complex to fabricate because they usually require high-temperature heating. In this research, a-C film as a temperature-sensitive material is chosen, and there are several advantages in terms of cost, power consumption, and process. First, the Pt noble metal target per gram is 600 times less expensive than a graphite target, resulting in low-cost a-C films [14,15]. Secondly, the temperature-sensitive film can be prepared at room temperature using magnetron sputtering or ion beam deposition methods. The process is simple, and the preparation cost of a-C temperature-sensitive material is lower than that of metal oxides. Thirdly, a-C film adheres very well to the substrate and does not need any metal transition layer. Last but not least, a-C film temperature sensors have low power consumption. Compared with a metal film temperature sensor, the resistance of a-C film is about 1000 ohms, according to P = U^2^/R, where U is the voltage value and R is the resistance of the temperature sensor thermistor; the larger R, the smaller the sensor power consumption. At present, the following studies are based on a-C temperature-sensitive films. T. Takeno et al. doped metals (W, Nb, and Cr) into diamond-like carbon (DLC) thin films by DC magnetron co-sputtering to study the temperature dependence of the film conductivity for different metal doping concentrations in the temperature range of 80~400 K [16]. We have also performed a preliminary study on a novel thermistor temperature sensor based on amorphous carbon (a-C) thin film prepared by an electron cyclotron resonance (ECR) plasma processing system. The sensor can operate over a wide temperature range of −75 to 155 °C with good linearity and repeatability [17]. In addition, a-C films [18,19,20,21] and hydrogenated amorphous carbon (a-C:H) [22,23,24,25,26] films are widely used as protective materials for their excellent properties, such as chemical inertness, high hardness, optical transparency, and low friction coefficient [27]. In addition, combined with their outstanding electrical properties and high-stress sensitivity [25,28], a-C films are a promising sensitive material used in MEMS sensors.

In this study, we proposed a simple preparation method to integrate the temperature sensor with the MEMS accelerometer. Here, we used the MEMS bulk micromachining technology to process the integrated sensor, in which the piezoresistive material of the accelerometer was prepared by ion implantation technology, and its sensitive structure was adopted as a cross-beam structure. The temperature-sensitive materials of the temperature sensor, a-C film, and a-C:H film, were prepared by DC magnetron sputtering and direct ion beam deposition, respectively. The surface morphology of the a-C thin film was characterized by SEM and TEM. Raman and XPS were used to analyze the states of sp^2^ and sp^3^ hybrid components. We performed the sensitivity tests on the temperature sensor, as well as the linearity, accuracy, and noise floor tests on the accelerometer. Finally, the output response of the integrated accelerometer was investigated at temperatures of 20~150 °C. The sensor showed excellent performance under actual test conditions.

## 2. Materials and Methods

### 2.1. Temperature-Sensitive Properties for a-C Thin Films

Before we fabricated the integrated sensors, we first studied the temperature-sensitive properties of amorphous carbon films under different fabrication processes. In this paper, a magnetron sputtering system (DISCOVERY635 Discovery, DENTON, Moorestown, NJ, USA) and an Ion Beam System are used to prepare amorphous carbon films. Then, we compared the temperature-sensitive properties of the two kinds of amorphous carbon.

The sample preparation procedure for studying the temperature-sensitive properties of a-C films is as follows. A 200 nm SiO_2_ film was covered on the surface of a 4-inch silicon substrate (p-type <100>) to obtain high electrical insulation, and an amorphous carbon film was subsequently deposited on it. The 4-inch wafer was then divided into 25 × 10 mm^2^ rectangles. Finally, we ultrasonically cleaned them with acetone and ethanol. 

We prepared amorphous carbon films using two methods. One was hydrogen-free amorphous carbon (a-C) films prepared by a DC sputtering system. The DC sputtering system produces a stable plasma with a high gas dissociation rate and energy coupling efficiency, usually without heating the substrate. The deposition rate can be controlled by the power and the bias voltage applied to the substrate. The system provides energy through 2.45 GHz microwaves. After the energy is absorbed by the gas (Ar), the gas is transformed into plasma. Therefore, we can adjust the content of sp^2^ and sp^3^ hybridized carbon atoms in the a-C film by changing the bias voltage. According to previous studies, the proportions between the two hybridized carbon atoms can affect the electrical properties of the film [17]. 

During the fabrication of a-C film, the pressure on the chamber during the vacuum was 3 × 10^−4^ Pa. The working pressure of the reaction chamber was 4 × 10^−2^ Pa through filling with Ar. Before the deposition of the a-C film, the substrate was cleaned by Ar^+^ ion sputtering for 3 min. Afterwards, the carbon target with the bias voltage of −300 V was bombarded with Ar^+^ ions. The substrate was applied with a +100 V bias voltage to generate low-energy electron irradiation during the deposition of the a-C film. The parameters of sputtering are shown in Table 1.

Alternatively, hydrogenated amorphous carbon films (a-C:H) were prepared by an Ion Beam System. In this paper, the work pressure of a-C:H thin film was 10^−3^ Pa, prepared by direct ion beam deposition. The gas used for the reaction was acetylene (C_2_H_2_), with a flow rate of 20 mL/min. When the vacuum of the chamber reached 2 × 10^−6^ Pa during the deposition process, a bias voltage was applied to the substrate so that the carbon-containing particles in the decomposition products were deposited onto the silicon substrate. Finally, the a-C:H thin film was prepared. The parameters of direct ion beam deposition are shown in Table 2.

**Figure 1:**
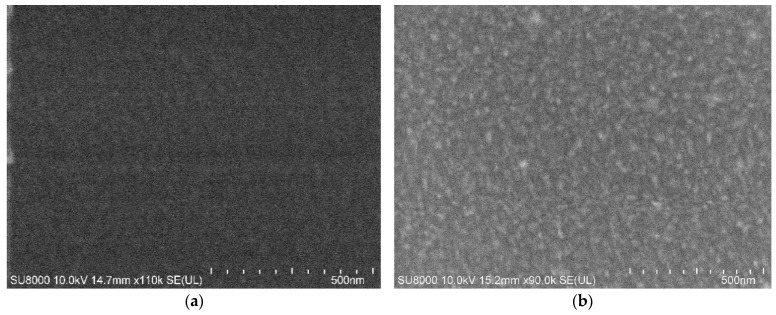
The SEM image of the amorphous carbon. (**a**) Prepared by DC sputtering; (**b**) prepared by ion beam.

Figure 1 shows the SEM image of the surface of the a-C and a-C:H thin film. The thickness of both thin films was 200 nm, measured by the Alpha-Step IQ stylus profiler. As seen in Figure 1a, the surface roughness of DC sputtering is less than that of ion beam deposition (Figure 1b). The surface of the DC sputtering amorphous carbon film is relatively uniform. In contrast, the surface of the ion beam deposited amorphous carbon film is relatively rough with uniformed clusters of about 30 nm.

**Figure 2:**
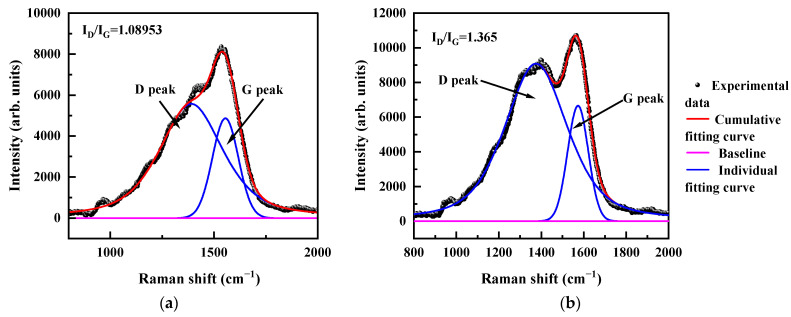
The Raman spectrum of the amorphous carbon. (**a**) Prepared by DC sputtering; (**b**) prepared by ion beam.

The temperature-sensitive amorphous carbon thin films were analyzed using Raman and XPS tests. In this paper, amorphous carbon films deposited by magnetron sputtering and linear ion beam were analyzed by Raman spectroscopy (Laser Raman Spectrometer, HORIBA, 532 nm, Kyoto, Japan). Figure 2 illustrates the Raman spectra of the amorphous carbon films deposited by magnetron sputtering and linear ion beam. All films showed typical asymmetric peaks of amorphous carbon in the wave number range of 800 cm^−1^ to 2000 cm^−1^. The Raman spectra could be fitted into the D and G peaks near 1360 cm^−1^ and 1550 cm^−1^, respectively, with two Gaussian fitting methods, where the D peak corresponds to the respiration vibration of sp^2^-C atoms in the ring structure, and the G peak corresponds to the telescopic vibration of sp^2^-C atoms in the ring and chain structures. As shown in Figure 1, here we observed asymmetric broad scattering Raman spectra centered over 1580 cm^−1^ and 1550 cm^−1^ in amorphous carbon films obtained by DC sputtering and linear ion beam deposition. An insignificant broad peak appears at 1390 cm^−1^ and 1300 cm^−1^. From the full width at half maximum of the G peak (FWHM), the peak area ratio of D peak to G peak (I_D_/I_G_), and the G-peak position, the size and distribution of the sp^2^ clusters can be qualitatively analyzed. In fact, I_D_/I_G_ is inversely proportional to the number of sp^2^ and clusters when the clusters of sp^2^ are larger than 2 nm., from which the degree of graphitization in the film can be calculated [29].

In Figure 2, the I_D_/I_G_ value of DC sputtering is 1.089, and that of the linear ion beam is 1.365. The SEM image shows that the particle size of the a-C:H film prepared by linear ion beam is larger than that of the a-C film prepared by DC sputtering. Since the a-C:H film has more defects, the ID/IG is large, indicating that the content of nanocrystalline graphene in the DC sputtered amorphous carbon film is higher than that in the ion beam amorphous carbon film because of the thermal decomposition of acetylene by the DC sputtered. Due to the weak adsorption at the adsorption boundary, the C–H bonds were broken in the film, unbonded hydrogen atoms were desorbed, and a large number of hydrogen atoms escaped from the film as hydrogen gas or C_x_H_y_. The dehydrogenated carbon structure underwent rearrangement driven by thermal energy and enhanced diffusion, and the broken sp^2^ bonds are interconnected to form more six-membered rings. This led to the increased ordering of the films and the increased content and size of nanocrystalline graphite clusters, indicating that the films’ graphite-like carbon features were enhanced.

**Figure 3:**
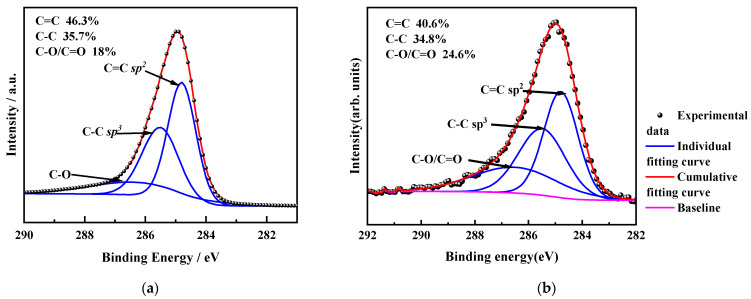
The XPS spectrum of the amorphous carbon. (**a**) Prepared by DC sputtering; (**b**) prepared by ion beam.

The chemical composition and chemical bonding of the amorphous carbon films were characterized using X-ray photoelectron spectroscopy (XPS, Axis UltraDLD, Kyoto, Japan); the films were first etched by Ar^+^ ion of 5 kV for 5 min to remove any oxides from the surface. The C 1s peaks were divided into three peaks by Gaussian (80%) and Lorentzian (20%) functions. The 284.6 eV, 285.4 eV, and 286.5 eV binding peaks shown in Figure 3 correspond to the sp^2^ hybridization (C=C), sp^3^ hybridization (C–C), and C–O/C=O hybridization, respectively. The content of sp^2^ hybridized carbon can be inferred by integrating the area of sp^2^ and sp^3^ hybridized carbon peaks. As shown in Figure 3, the content of C=C in the DC sputtered carbon film is 46.3%, while that in the ion beam deposited carbon film was 40.6%, much lower than that of DC sputtering. It also indicates that the content of sp^2^ hybridized carbon bond in the ion beam carbon film is lower than that in the DC sputtering. The C–C content of DC sputtering is 35.7%, and the C–O/C=O content is 18%. For the film deposited by the linear ion beam, the C–C content is 34.8%, and the C–O/C=O content is 24.6%. Both types of carbon films have higher sp^2^ content than sp^3^ content inside.

**Figure 4:**
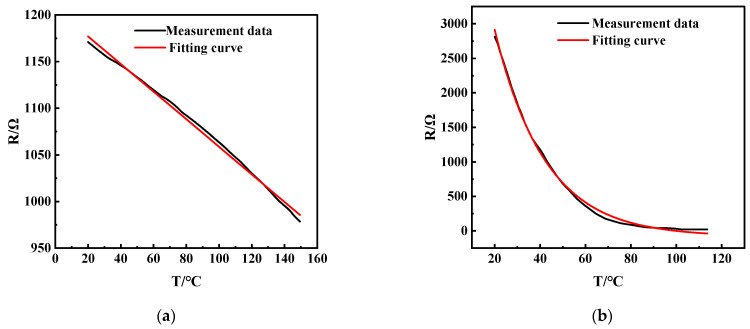
Test results of temperature-resistance characteristics of amorphous carbon thin films. (**a**) Prepared by DC sputtering (Sample A); (**b**) prepared by ion beam (Sample B).

We used the oven (ESPEC PG-2J, Osaka, Japan) for temperature testing with a temperature fluctuation range of ±0.3 °C, which was carried out in the programmed operation mode, and the slope control was applied to the temperature rising process. The multimeters (FLUKE 8846A, Everett, WA, USA) were used to record the data. Figure 4a shows the test results of sample A, which has good linearity in the range of 20 °C~150 °C. The temperature coefficient of resistance (TCR) of the temperature sensor was 1.28 × 10^−3^/°C. After fitting, the relationship between the resistance (*R*) and the temperature (*T*) can be fitted as follows:

(1) R=−1.5T+1206.1

Figure 4b shows the test results of Sample B. The general trend of the resistance decreases with increasing temperature, but it fluctuates significantly in the range of 20–60 °C, which is unfavorable for practical applications. After the exponential fitting, the approximate relationship between the resistance (*R*) and the temperature (*T*) of Sample B can be obtained as follows:

(2) $$R=-91.24+7302.8e^{-0.044T}$$

Overall, the resistance (*R*) of the amorphous carbon film shows a negative correlation with the temperature (*T*). It is known that the electrical properties of the amorphous carbon film are mainly affected by the sp^2^ hybridized carbon content. However, the change of H content greatly impacts the sp^2^ clusters, which takes work to control. As the temperature monotonically varies with the resistance, the amorphous carbon film deposited by the linear ion beam (a-C:H) can be used as a temperature sensor. However, the DC magnetron sputtered carbon film is more suitable as a temperature sensor because of the better linearity of the temperature versus resistance curve.

### 2.2. Temperature-Sensitive Mechanism of a-C Thin Films

**Figure 5:**
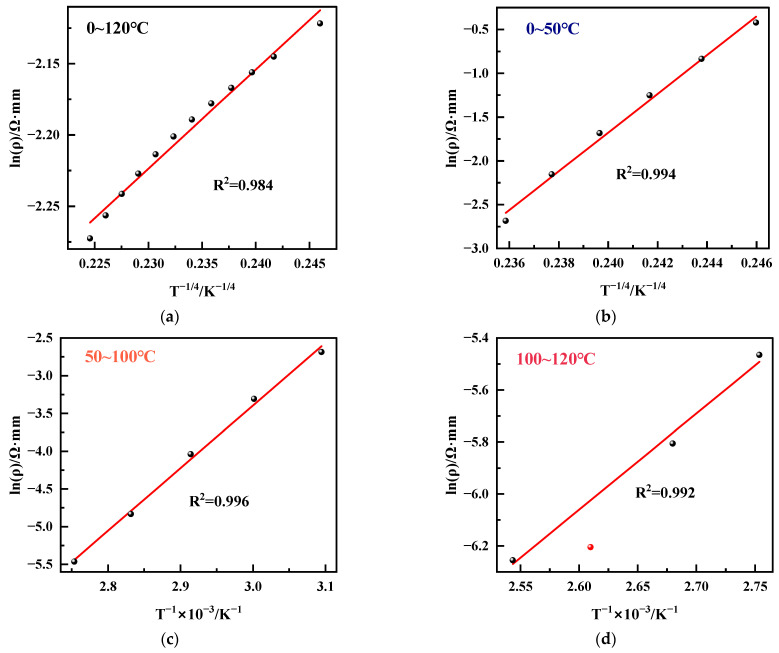
Temperature dependence of electrical conductivity of a-C and a-C:H films. (**a**) Prepared by DC sputtering (Sample A); (**b**) prepared by ion beam (Sample B) at the temperature range of 0~50 °C. (**c**) Prepared by ion beam (Sample B) at the temperature range of 50~100 °C. (**d**) Prepared by ion beam (Sample B) at the temperature range of 100~120 °C.

As shown in Figure 5, we fitted the relationship between resistivity *ρ* and temperature *T*, which allows us to analyze the mechanism of carrier transport in the material. Unlike crystalline semiconductors, the localized electrons in amorphous semiconductors can only be conducted by tunneling or thermal activation [30]. The resistivity-temperature curve of an amorphous semiconductor is related as follows [31]:

(3) $$\rho =\rho_{0}\exp{\left(T_{0}/T\right)}^{n}$$

where *ρ* is the resistivity, *T* is the test temperature, and *ρ*_0_ and *T*_0_ are both constants. *n* takes the values 1, 1/4, and 1/2, whose carrier transport behavior corresponds to thermal activation, three-dimensional Mott variable range hopping conduction, and variable range hopping conduction in the Coulomb gap, respectively.

From the analysis of the XPS data in Figure 3, it can be obtained that the content of sp^2^ clusters in the a-C films prepared by DC magnetron sputtering is more than that in the a-C:H films deposited by ion beam, and the carriers are dominated by three-dimensional Mott variable range hopping conduction between the sp^2^ clusters due to the closer sp^2^ cluster spacing. The linear ion beam deposited amorphous carbon film with less sp^2^ content is dominated by three-dimensional Mott variable range hopping conduction at room temperature or low temperature, and when the temperature is greater than 50 °C, due to the increase of cluster spacing, the variable range jump probability decreases, and thermal diffusion conduction is dominated at this time. As shown in Figure 5a, for the DC-sputtered a-C material (Sample A), ln(ρ) is linearly related to T^−1/4^ in the range of 0~120 °C, and the corresponding carrier transport mode is three-dimensional Mott variable range hopping conduction. Since there is no change in the carrier transport in the range of 0~120 °C, the DC sputter-deposited a-C film is used as a temperature-sensitive material with better linearity. As for the ion beam deposited a-C:H film (Sample B), ln(ρ) is linear with T^−1/4^ in the range of 0~50 °C, and ln(ρ) is linear with T^−1^ in the range of 50~120 °C. Thus, the a-C:H film is three-dimensional Mott variable range hopping conduction in the low-temperature region and thermally activated conduction in the high-temperature region. This is because thermally activated band conduction does not easily occur within the sp^3^ clusters in a-C:H at low temperatures. The conductivity in the low-temperature region decreases sharply with increasing temperature. The carrier transport is affected by the size and number density of the sp^2^ clusters as well as the dangling bonds at the cluster interface. When the temperature is greater than 50 °C, the carrier transport mechanism changes from VRH conduction to thermally activated conduction. The change in the carrier transport mechanism leads to poor linearity of the a-C:H films deposited by an ion beam.

### 2.3. Design and Fabrication of Temperature Sensor

**Figure 6:**
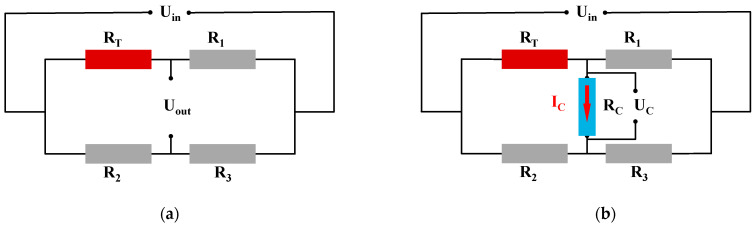
(**a**) Theoretical Wheatstone bridge circuit for temperature sensors; (**b**) actual Wheatstone bridge circuit for temperature sensors.

Our sensor is a Wheatstone bridge temperature sensor; the input to the circuit is a voltage, and the output voltage is proportional to the input voltage. In the Wheatstone bridge of Figure 6, *R*_1_, *R*_2_, and *R*_3_ are known, and *R_T_* is the unknown temperature resistance. When the potential (voltage) P_1_ is the same as the potential P_2_, the bridge is said to be balanced. In this condition, no current outputs and the ratio of resistance in the *R_T_*-*R*_2_ path must equal the ratio in the *R*_1_-*R*_3_ path. For measuring temperature, a Wheatstone bridge is used in out-of-balance where the out-of-balance voltage, Δ*U*, can be measured and related to the resistance of the thermistor. See the simple DC bridge circuit, as shown in Figure 5, that is used for such precision measurement using the thermistor. A correct choice of resistors *R*_2_ and *R*_3_ will remove the mean DC value of Δ*U*. In general,

(4) $$U_{\mathrm{out}}=U_{in}\left(\frac{R_{2}}{R_{3}+R_{2}}-\frac{R_{T}}{R_{1}+R_{T}}\right)$$

Assume *R*_1_ = *R*_3_ = *R*_2_ = *R_T_*_0_ = *R*, where *R_T_*_0_ is the initial state of temperature sensor resistance. Then,

(5) $$U_{out}\approx \frac{1}{4}\frac{\Delta R}{R}U_{in}$$

**Figure 7:**
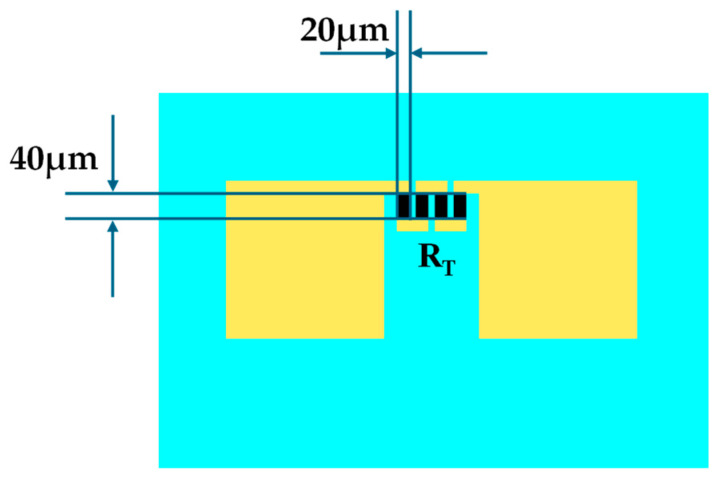
Design parameters of a-C temperature sensor.

The actual circuit of the temperature sensor is shown in Figure 6b, which is calculated as follows:

(6) $$U_{C}\approx U_{out}\frac{R_{C}}{R+R_{C}}$$

(7) $$I=\frac{U_{in}}{2R}$$

where *U_C_* is the output load voltage (the actual output voltage), *R_C_* is the output load, and *I* is the current flowing through the temperature sensor bridge leg. From Equation (6), when R is too large, U_C_ deviates too much from the ideal value. As can be seen from Equation (7), when R is too small, the current flowing through the bridge leg is too large, and power consumption increases. Therefore, the bridge leg resistance of a-C temperature-sensitive material is 2~5 kΩ, and the current is below 1~2 mA. Due to the resistivity of the a-C film is 0.01Ω∙cm, to ensure that the resistor resistance is below 4 kΩ, the length of the resistor is eight times the width, which takes up more space. As shown in Figure 7, the resistor is therefore designed as a quadruple-fold resistor strip, and the size of each fold of resistor strips is 40 × 20 μm (l × w).

**Figure 8:**
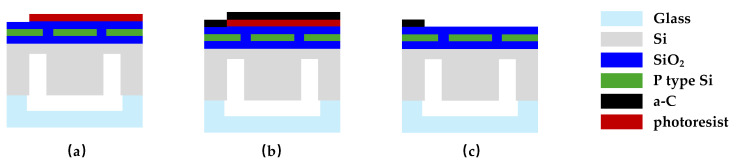
Temperature sensor processing flow diagrams. (**a**) Photolithography; (**b**) a-C deposition; (**c**) lift off.

The proposed a-C temperature sensor was fabricated based on DC sputtering with the graphite target of at least 99.999% purity and the lift-off feature definition process. Prototypes were fabricated based on the substrate of 4-inch single-crystal silicon wafers with natural surface oxidation. The fabrication processing flow of the proposed a-C-film temperature sensor is illustrated in Figure 8. The details of the processes are as follows: (1) the surface of the silicon wafer was cleaned by organic solutions and plasma cleaning to remove impurities and organic substances; (2) the disc was uniformly spin-coated with double-layer photoresist (AZ4620). Photolithography was used to define the patterns and transfer the patterns by NaOH developer; (3) the DC magnetron sputtering was used to deposit a-C thin film with a thickness of 200 nm on the substrate surface; (4) the sample wafer was immersed in acetone to remove AZ4620. In this case, the a-C thin film on the photoresistor could be lifted off, leaving the required features on the substrate. 

### 2.4. Design and Simulation Optimization of Accelerometer Piezoresistive Sensitive Structure

**Figure 9:**
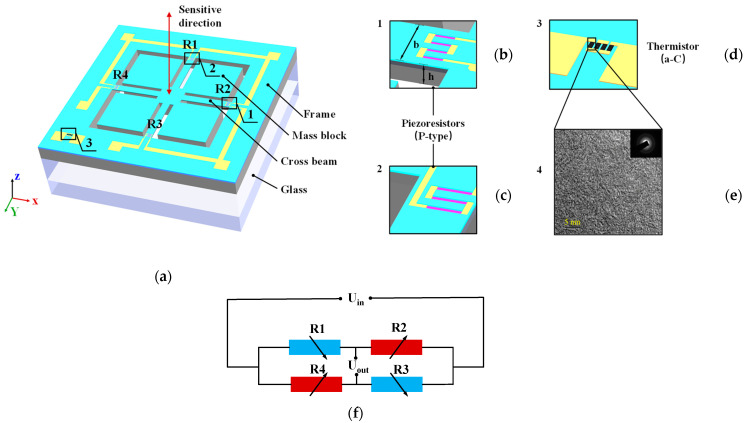
(**a**) Schematic of accelerometer; (**b**) zoomed-in view of R_2_; (**c**) zoomed-in view of R_1_; (**d**) zoomed-in view of temperature sensor; (**e**) TEM picture of the surface of the a-C temperature sensor. (**f**) read-out circuit.

The accelerometer was designed based on the piezoresistive effect of P-type Si. Since P-type silicon has a larger piezoresistive coefficient than other piezo-sensitive materials and higher linearity compared to N-type silicon, the accelerometer in this paper adopted the P-type doping process to fabricate the piezoresistor. Moreover, the resistors were arranged along the [110] orientation at (100) plane to obtain the maximum piezoresistive coefficient and improve the measurement sensitivity of the sensor. The longitudinal piezoresistive coefficient is 71.8 × 10^−11^ m^2^/N, and the transverse piezoresistive coefficient is −66.3 × 10^−11^ m^2^/N [9,10]. The structure of the sensor is shown in Figure 9a. The sensitive structure of the silicon accelerometer in this study utilized an embedded cross-beam structure, with a proof mass connected to the fixed frame through the beam. In order to improve the sensitivity of the sensor, four beams were extended into the mass block to increase the length of the beams. Four piezoresistors are arranged on the four beams near the root of the frame. When power is applied, the current direction on *R*_1_ (Figure 9c) and *R*_3_ are perpendicular to the strain direction, while the current direction on *R*_2_ (Figure 9b) and *R*_4_ are parallel to the strain direction. According to the piezoresistive effect of P-type Si, the resistance changes caused by stress on the four beams can be calculated as follows:

(8) $$\frac{\Delta R_{i}}{R_{i}}=\pi_{t}\sigma_{R_{i}}(i=1,3)\;\frac{\Delta R_{i}}{R_{i}}=\pi_{l}\sigma_{R_{i}}(i=2,4)$$

where Δ*R*_1_, Δ*R*_2_, Δ*R*_3_, and Δ*R*_4_ are the resistance changes of *R*_1_, *R*_2_, *R*_3_, and *R*_4_, *π*_l_ and *π*_t_ are the longitudinal and transverse piezoresistive coefficients of P-type Si, respectively, *σ_R_* is the acceleration-induced stress on the piezo resistor R. Owing to the positive transverse piezoresistive coefficients and the negative longitudinal piezoresistive coefficients of P-type Si, the resistances changes under strain are opposite. The read-out circuit is shown in Figure 9d.

**Figure 10:**
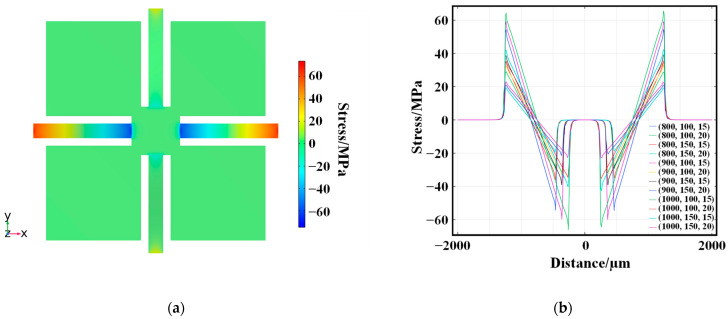
(**a**) Finite element simulation analysis of embedded cross-beam sensitive structures; (**b**) Stress simulation of sensitive beams with different sizes of accelerometer.

When the input acceleration is 80 g, the result of stress distribution is shown in Figure 10a. From the stress distribution, the beams have the most significant stresses at the root close to the fixed frame. Therefore, the piezo resistors are placed on four sensitive beams close to the fixed frame, which form a Wheatstone full bridge to improve the output sensitivity of the circuit.

The output of the accelerometer is calculated as follows:

(9) $$U_{o}=\frac{\Delta R}{R}U_{i}$$

where $U_{o}$ is the output of the sensor, $U_{i}$ is the supply voltage (normally 5 V). The change of output $U_{o}$ can reflect the magnitude of acceleration.

The length, width, and thickness of the beam are essential influences on the maximum stress of the sensitive structure, and parametric scanning is required to determine the optimal structural dimensions. The finite element simulation results under multiple dimensions are shown in Figure 10b. The x-direction stress distribution on the upper surface of the four-beam sensitive structure is significantly affected by the dimensions of the sensitive beam. Due to the symmetry of the structure, there is no need to discuss the stress distribution in the y-direction. From Figure 10b, the stress at the root of the beam is maximum when the beam size (*l* × *b* × *h*) is 1000 μm × 150 μm × 15 μm.

### 2.5. Integrated Model of a-C Temperature Sensor and MEMS Accelerometer

**Figure 11:**
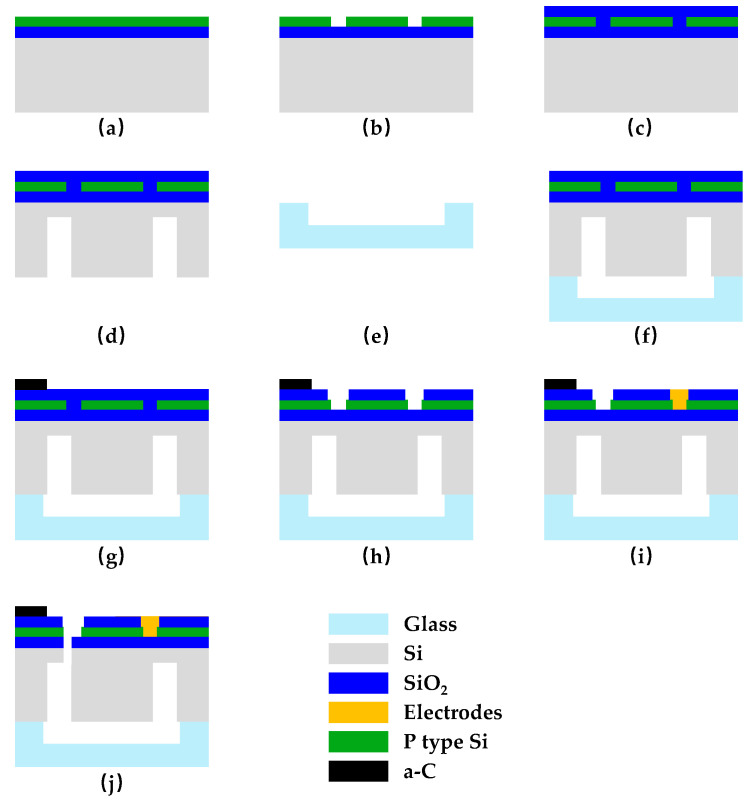
Integrated temperature sensor and accelerometer processing flow diagrams. (**a**) Ion injection; (**b**) resistor etching; (**c**) deposited insulation layer; (**d**) back cavity etching; (**e**) glass corrosion; (**f**) silicon-glass bonding; (**g**) thermistor deposition; (**h**) metal wire etching; (**i**) electrode sputtering; (**j**) front structure release.

Figure 11a shows a schematic of the MEMS accelerometer chip with a four-cross beam-mass structure used in this work. The Silicon accelerometer integrates with the amorphous carbon (a-C) thin film temperature sensor (Figure 9d), and the four doped piezo resistors are located at the root of the four beams. The temperature resistance is deposited on the fixed silicon frame of the chip by magnetron sputtering. A quad-fold form layout is used to increase the resistance value of the temperature resistor.

In this paper, the amorphous structure inside the amorphous carbon film was characterized using a high-resolution transmission electron microscope (JEM-2100, JEOL Ltd., Tokoyo, Japan, accelerating voltage 200 keV) in combination with selected-area electron diffraction, as shown in Figure 9e. The amorphous carbon film prepared by magnetron sputtering produced the nanocrystalline structure of graphene.

### 2.6. Fabrication of Integrated Temperature Sensor and Accelerometer

We used 4-inch double-sided polished SOI wafers to process the devices, and the thicknesses of the device layer, BOX layer, and handle layer were 1.2 µm, 400 nm, and 500 µm, respectively. As shown in Figure 11a, ion injection of the SOI device layer was carried out with an injection dose of 1.2 × 10^16^ cm^−2^ and an injection energy of 80 keV. To ensure the piezo resistor reached the desired and uniform impurity concentration, we used a 1.5 h 1100 °C high-temperature annealing process. At that time, the square resistance of the piezo resistor was about 10 Ω/□. The piezo resistor strip was patterned by an ICP (induced coupled plasm) etching process, as shown in Figure 11b. Figure 11c shows the deposition of a 500 nm-thick silicon oxide, the insulating layer, on the surface of the device layer. Figure 11d shows patterning and deep-silicon dry etching of the SOI handle layer by photolithography and the ICP etching process to develop a 470 µm deep back cavity. To limit the displacement of the mass block to prevent the sensitive beam from breaking after overloading and to facilitate subsequent processing, we patterned the BF33 glass substrate through a wet corrosion process to form a 200 µm deep groove in Figure 11e. Figure 11f shows silicon-glass bonding, where the SOI handle layer is bonded to the BF33 glass to form a good seal at the silicon-glass interface. As shown in Figure 11g, amorphous carbon thermistor strips were deposited and patterned into the surface of the insulation layer. After that, the metal electrode was sputtered to form the electrical connection between the temperature sensor and the accelerometer (Figure 11h), where the metal electrode material was 30 nm thick Cr and 100 nm thick Pt. Finally, the front structure of the sensor was etched by the ICP etching process to form the mass block and the sensitive beam (Figure 11j). 

## 3. Measurement of the Sensor

### 3.1. Temperature Testing of Temperature Modules with Integrated Sensor

**Figure 12:**
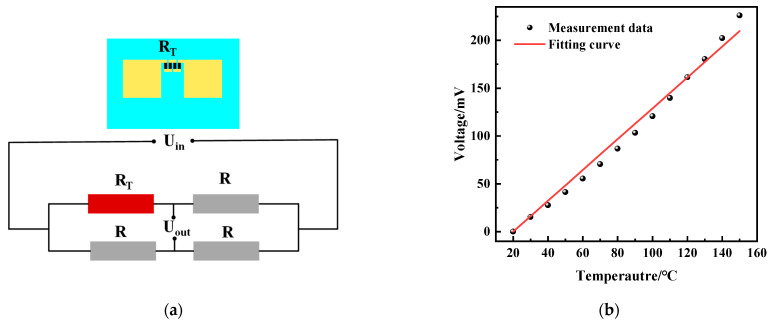
Test results of a-C temperature sensor modules with integrated sensor. (**a**) Read-out circuit of temperature sensor modules; (**b**) the output voltage versus temperature of temperature sensor modules.

We placed the integrated sensors in the oven to test the temperature characteristics. The temperature-sensitive resistor was connected to the Wheatstone circuit shown in Figure 12a, and the other three legs of the Wheatstone bridge were adjusted so that their resistance was equal to that of the thermistor of the integrated sensor in the initial state. The circuit was supplied with a voltage of 5 V, and the output voltage was 0 mV in the initial state. Figure 12b shows the output voltage versus temperature of the temperature modules of the integrated sensor. The sensor was in the range of 20~150 °C. Since the quad-folded a-C thermistor had a large resistance (of approximately 4.7 kΩ), the current of each leg in the Wheatstone bridge was 0.5 mA. Meanwhile, comparing the external temperature, the power consumption of the sensor was sufficiently low so that the self-heating effect was negligible. By the linear fitting of this output–temperature relationship, the sensitivity, and linearity of the sensor were obtained. The linearity is expressed by the coefficient of determination (R^2^) of the fitted line. The sensor has good linearity in the range of 20 to 150 °C, the sensitivity is 1.62 mV/°C with the input voltage of 5 V, and the linearity is 0.99578. The temperature module of this integrated sensor has better linearity and higher sensitivity than other temperature sensors (Table 3).

### 3.2. Static Testing of Accelerometer Modules with Integrated Sensor

**Figure 13:**
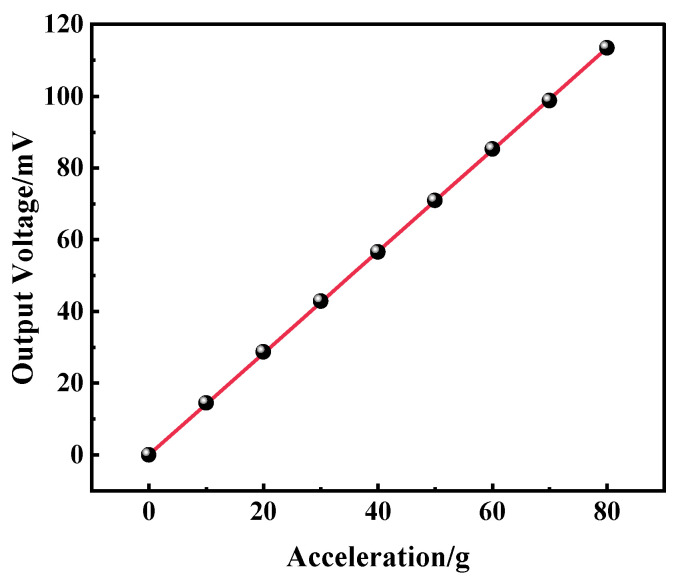
Static test results of the accelerometer.

The centrifuge system used in this paper is the SY30-3 centrifugal constant acceleration tester developed by Suzhou Dongling Vibration Testing Instrument Co., Ltd. (Suzhou, China). The tester consists of the control mainframe and the working rotary table. The main structure of the SY30-3 rotary table steady-state acceleration tester (centrifugal machine) consists of a speed measurement system, a motor, a speed reducer, a working rotary table, and an electrically conductive slip ring. The centrifuge system can apply a maximum acceleration of 80 g, and the minimum acceleration interval is 3 g. During the experiment, the control host generated the control signal to drive the rotary table to rotate, thus generating the acceleration signal. The sensor in the system is powered by a DC power supply with an excitation voltage of 5 V; the output of the sensor is detected by a desktop multimeter. The acceleration test range of the experiment is 0–80 g. The sensitivity of the accelerometer can be obtained from Figure 13 as 1.4 mV/g/5 V. The linearity is 0.38%, and the repeatability is 1.56%.

**Figure 14:**
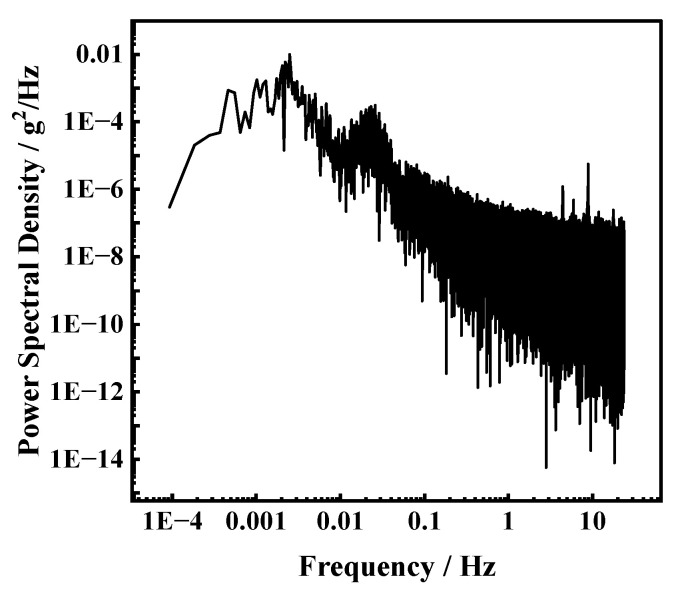
The noise power density spectrum of accelerometer modules of integrated sensor.

In order to evaluate the acceleration measurement resolution of the accelerometer, a noise floor evaluation experiment was conducted. The sensor was placed in a laboratory environment for 3 h with a sampling rate of 208 Hz. The noise power density spectrum (PSD) is shown in Figure 14, which shows that the average power per unit of noise frequency fluctuates between 10^−13^ and 10^−6^ g^2^/Hz when the vibration frequency of the integrated sensor is greater than 1 Hz. The total thermomechanical noise in the range of 1~20 Hz is 509 µg, and the average thermomechanical noise density is 116 µg/$\sqrt{\mathrm{Hz}}$, they are two to three magnitudes smaller than the input acceleration amplitude for testing sensitivity. Therefore, they do not significantly affect the sensor measurements or the derived performance metrics.

### 3.3. Temperature Effects on Accelerometers of Integrated Sensor

**Figure 15:**
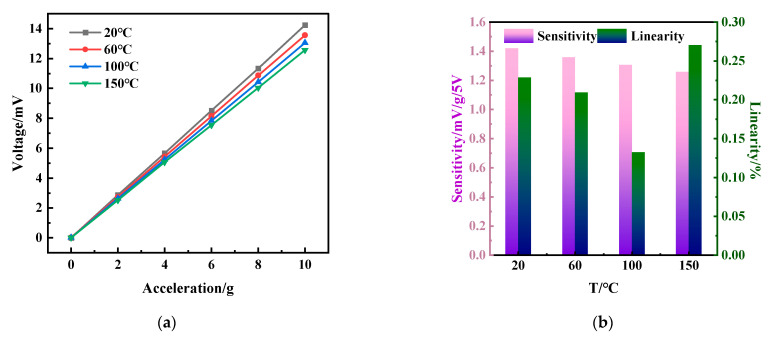
Temperature effect on the accelerometer of the integrated sensor. (**a**) The output voltage versus acceleration in the temperature range of 20 °C to 150 °C; (**b**) the sensitivity and linearity of the accelerometer in the temperature range of 20 °C to 150 °C.

Figure 15 shows the output of the integrated sensor accelerometer module over the range of 20 °C to 150 °C. The integrated sensor was connected to the protruding end of the vibration shaker (JZK-5, Chengtec, Shanghai, China) via a stainless rod, and the sensor was placed in a temperature chamber (STH-120, Espec, Japan) through the protruding end, where the temperature chamber can be manually controlled to generate a stable temperature environment within 200 °C, with a temperature distribution variation of less than 2.5 °C in the chamber. The vibration shaker can provide acceleration up to 10 g at the operating frequency, controlled by a function generator. The sinusoidal signal from the function generator is amplified by a power amplifier (GF-20) and then introduced into the vibration shaker to generate the desired vibration signal. The signal from the sensor is subsequently digitized with a DASP signal acquisition system and displayed on a laptop computer. Subsequently, we processed the acquired data by Fast Fourier Transform (FFT) to obtain the RMS value of the sensor’s output at a constant vibration frequency. In addition, a commercial vibration sensor (CK8305) is used as a reference to calibrate the acceleration at room temperature. In our experiments, we used a constant vibration frequency of 160 Hz with an input acceleration in the range of 0~10 g at 20~150 °C. The acceleration frequency of 160 Hz was used because this frequency is sufficiently high to obtain relatively low 1/f noise and is sufficiently close to the commonly used frequency for acceleration calibrators; otherwise, at this frequency, the commonly known noise source at 50 Hz and its multiples can be avoided. As shown in Figure 15a, the acceleration sensitivity of the integrated sensor obtained by the fitting method is 1.42 mV/g/5 V, which is only a 1.4% discrepancy compared to the centrifuge test result. As the temperature increases (Figure 15b), the sensitivity of the sensor decreases to 1.36 mV/g/5 V at 60 °C, 1.31 mV/g/5 V at 100 °C, and 1.26 mV/g/5 V at 150 °C. The calculated temperature coefficient of sensitivity (TCS) of the sensor is 0.087% FSO/ °C. The decrease in sensitivity of the sensor is mainly due to the decrease in the piezoresistive coefficient of P-type silicon with increasing temperature. The linearity of the sensor is less than 0.27% over the temperature range of 20 to 150 °C. The integrated sensor’s high sensitivity, excellent linearity, and low-temperature coefficient allow it to be used for temperature and acceleration testing in the temperature range of 20~150 °C.

### 3.4. Temperature Compensation on Accelerometers of Integrated Sensor

Since the sensitivity of the acceleration part of the integrated sensor varies linearly with temperature, the temperature sensitivity coefficient TCS of the integrated sensor is 0.087% FSO/°C. In order to minimize the effect of temperature on the output of the accelerometer, a temperature compensation method based on the least squares method is used to convert the sensor at different temperatures to a value at room temperature. The output of the accelerometer can be expressed at different temperatures as follows:

(10) $$U(T)=S(T)\cdot a+U_{Z}(T)$$

where *U*(*T*) is the sensor output, *S*(*T*) is the sensor sensitivity, and *U_Z_*(T) is the zero bias of the sensor at temperature *T*; *S*(*T*) decreases as the temperature increases from 20 to 150 °C. According to (5), the temperature compensation is expressed as follows:

(11) $$U_{C}(T)=S_{0}\frac{U(T)-U_{Z}(T)}{S(T)}\cdot a+U_{Z0}$$

where *U_C_*(*T*) is the sensor output after compensation, *S*_0_ and *U_Z_*_0_ are the sensitivity and zero-offset of the sensor at room temperature.

**Figure 16:**
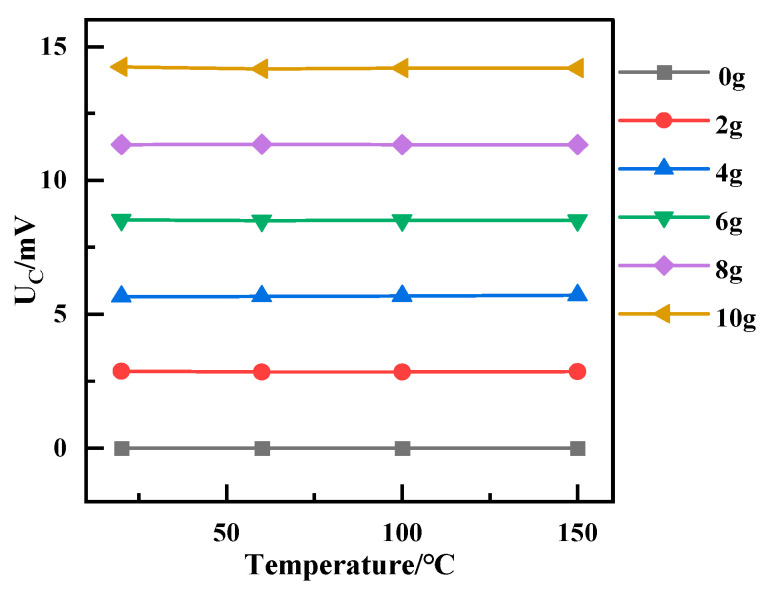
Sensor output after temperature compensation.

Figure 16 shows the results of temperature compensation; at different temperatures, the output of the compensated temperature sensor remains stable, and the effect of temperature on the accelerometer is reduced.

## 4. Conclusions

In this paper, a temperature sensor integrated with a MEMS accelerometer was reported. The thermal resistive material for the temperature sensor is an amorphous carbon thin film, and the piezoresistive material for the accelerometer is P-type silicon. The structure of the accelerometer was the four-cross beam-mass structure, and the simulation and optimization of its structure were performed to determine the final size under the overload of 80 g (1000 μm × 150 μm × 15 μm). The amorphous carbon film was deposited by DC magnetron sputtering and linear ion beam, respectively. Then, we characterized the two types of film by SEM and TEM. The ratio of I_D_/I_G_ and the cluster size of sp^2^ were analyzed by Raman, and the content of sp^2^ and sp^3^ of the carbon film was analyzed by XPS, which elucidated the relationship between the size and content of sp^2^ cluster and the temperature-sensitive properties of amorphous carbon films. It indicated that the DC-sputtered amorphous carbon films had better linearity and were more suitable for preparing temperature sensors. Finally, the proposed accelerometer integrated with a temperature sensor chip was designed and processed. The temperature sensor module has a sensitivity of 1.62 mV/°C (the input voltage of 5 V) with a linearity of 0.99578 in the temperature range of 20~150 °C. The sensitivity of the sensor is slightly higher than that of traditional metal film temperature sensors. The accelerometer module has a sensitivity of 1.4 mV/g/5 V, a nonlinearity of 0.38%, a repeatability of 1.56%, a total thermomechanical noise of 509 µg over the range of 1 to 20 Hz, and an average thermomechanical noise density of 116 µg/√Hz, which is smaller than the input acceleration amplitude for testing sensitivity. The temperature performance of the sensor was verified. At 150 °C, the sensitivity of the sensor was 1.26 mV/g/5 V, and the temperature coefficient of sensitivity (TCS) was 0.087% FSO/°C. This work contributes to the broader application of a-C films in MEMS sensors and a more cost-effective, efficient, and environmentally friendly manufacturing process for piezoresistive components.

## Figures and Tables

**Table 1:** The parameters of DC sputtering.

Process Parameters	Values
Sample ID	A
Method	DC Sputtering
Substrate Bias (V)	+100
Temperature (°C)	200
Time (min)	30
Thickness of film (nm)	200

**Table 2:** The parameters of direct ion beam deposition.

Process Parameters	Values
Sample ID	B
Method	Ion Beam
Substrate Bias (V)	+1300
Temperature (°C)	200
Time (min)	125
Thickness of film (nm)	200

**Table 3:** Different temperature sensors.

Materials	Sensitivity (mV/°C)	Range (°C)	Linearity	Input Type	Ref.
TiN/GaN SBD	0.9~1.6	25~200	0.9987	10^−5^~0.1 (A)	[32]
AlGaN/GaN	−6.9	25~275	/	12 (V)	[33]
Pt	0.00194/°C	25~700	/	/	[34]
PdNi	0.00122/°C	31~57	/	/	[35]
Au	0.0032/°C	27.5~32.5	0.99998	/	[36]
InP	−1.1~2.1	16~60	/	12 (µA)	[37]
Pt/Pt-13Rh	0.01	300~800	0.99	111 pairs	[38]
This work	1.62 1.28 × 10^−3^/°C	20~150	0.99578	5 (V)	

## Data Availability

The experimental data is private, so please contact us if the authors would like it.
